# Experiences of music therapy in paediatric palliative care from multiple stakeholder perspectives: A systematic review and qualitative evidence synthesis

**DOI:** 10.1177/02692163241230664

**Published:** 2024-03-07

**Authors:** Victoria Kammin, Lorna Fraser, Kate Flemming, Julia Hackett

**Affiliations:** The Paediatric Palliative Care Research Group, Department of Health Sciences, University of York, York, UK

**Keywords:** Paediatric palliative care, music therapy, child, family, qualitative research, systematic review

## Abstract

**Background:**

Children and young people with life-limiting conditions and their families need physical and emotional support to manage the challenges of their lives. There is a lack of synthesised qualitative research about how music therapy is experienced by children, young people and their families supported by paediatric palliative care services.

**Aim:**

To systematically identify and synthesise qualitative research on experiences of music therapy in paediatric palliative care from stakeholder perspectives.

**Design:**

A Qualitative Evidence Synthesis was conducted using Thematic Synthesis. The review protocol was registered in PROSPERO (registration number: CRD42021251025).

**Data sources:**

Searches were conducted with no dates imposed via the electronic databases PsycINFO, MEDLINE, EMBASE, AMED and CINAHL in April 2021 and updated in April 2022. Studies were appraised for quality using the Critical Appraisal Skills Programme tool (CASP).

**Results:**

A total of 148 studies were found, 5 studies met the eligibility criteria reporting the experiences of 14 mothers, 24 family members and 4 staff members in paediatric palliative care. There were five overarching themes: emotional and physical reprieve, opportunity for normalised experiences, thriving despite life limited condition, enhance family wellbeing and therapeutic relationship central to outcomes.

**Conclusion:**

Music therapy provides unique benefits for this paediatric population particularly in supporting child and family wellbeing. The therapeutic relationship, interpersonal skills of the therapist and experience in paediatric palliative care are perceived as central to these positive outcomes.

**What is already known about the topic?**

-  Whilst the benefits of music therapy in paediatric palliative care are recognised, provision varies considerably within and across countries.
-  Stakeholder experiences of music therapy in paediatric palliative care are under-represented in existing literature and therefore do not contribute to service development.
-  There is no international review of experiences of music therapy in paediatric palliative care.

**What this paper adds?**

-  Music Therapy provides unique benefits in paediatric palliative care, particularly in supporting child and family wellbeing.
-  The therapeutic relationship, interpersonal skills of the therapist and experience in paediatric palliative care are central to positive outcomes.
-  Evidence for funding purposes, development of provision and successful recruitment.

**Implications for practice, theory or policy**

-  Paediatric palliative care settings should strive for music therapy provision for children and families using their services to support child and family wellbeing.
-  Music therapists should be recruited with interpersonal skills and experience as central to the recruitment process and provided with ongoing training and support.
-  Further high-quality research that captures the voices of the child and family articulating their own experiences of music therapy is recommended, positioning these voices as central to service development and provision.

## Background

The World Federation of Music Therapy define music therapy as the ‘professional use of music and its elements as an intervention in medical, educational and everyday environments with individuals, groups families or communities who seek to optimise their quality of life and improve their physical, social, communicative, emotional, intellectual and spiritual health and wellbeing’ (https://www.wfmt.info/). Research indicates that goals outlined by the World Health Organisation for palliative care can be addressed through music therapy, including reduction of anxiety, pain, increasing emotional expression and improving family interactions.^1^ However, current music therapy literature in palliative care is predominantly focussed on adult palliative care, bereavement, end of life or oncology patients.^2–7^ There is an increasing prevalence of life-limiting conditions in children and young people rising from 26.7 per 10,000 to 63.2 per 10,000 between 2001 and 2018 in England,^8^ whilst there is relatively limited evidence for music therapy for children with life-limiting conditions.^2–7^

It is important that we optimise research that is available to inform the development of music therapy provision in paediatric palliative care from all stakeholder perspectives. This will help ensure recognition of music therapy beyond an optional addition to paediatric palliative care services, enabling its value to be evidenced for future development and funding purposes. There is a body of qualitative research which has explored stakeholders’ experiences of music therapy in paediatric palliative care, however this has not ever been synthesised. Doing so will enable a deeper understanding of how music therapy is experienced by, and its potential value to, children and young people with life limiting illnesses and their families and support future service development.

Qualitative Evidence Synthesis is a systematic review methodology for the synthesis of primary qualitative research. Qualitative Evidence Synthesis enables the dev-elopment of new and more in depth understanding and interpretation than is possible from single studies alone.^9^ Flemming et al.^10^ report that the findings from Qualitative Evidence Synthesis can ‘enable a greater understanding of individuals’ and groups’ experiences, views, beliefs and priorities for healthcare’, therefore Qualitative Evidence Synthesis is a methodology highly appropriate to this study. The overall aim of this Qualitative Evidence Synthesis was to identify and synthesise qualitative research related to stakeholder experiences of music therapy in paediatric palliative care in order to understand these experiences to inform future service development.

## Methods

A Qualitative Evidence Synthesis was conducted using Thematic Synthesis, an approach to synthesising qualitative research papers.^11^ Thematic synthesis^11^ was chosen for its accessibility, capacity to synthesise diverse studies from various epistemological standpoints and suitability for investigating under-researched areas. Thematic Syn-thesis is an established methodological approach to synthesising qualitative research and is one of the core Qualitative Evidence Synthesis methodologies advocated by the Cochrane Qualitative and Implementation Methods Group (CQIMG).^12^ In order to undertake this review according to the most contemporaneous Qualitative Evidence Synthesis methods, we drew on the key components of Thematic Synthesis, that is, line by line coding and development of descriptive and analytical themes, however looked to more recent literature to guide our approach to other areas of Qualitative Evidence Synthesis processes, for example, searching and critical appraisal.

This was employed by extracting data from each study beginning with the line-by-line coding of the findings of the individual qualitative studies to identify areas of similarity that have the potential to be developed into descriptive themes. The synthesis aimed to go beyond these descriptive themes to develop analytical themes in order to generate new constructs, explanations or hypotheses.^13^ The coding process was conducted by a single reviewer with regular communication with the review team during the development of descriptive and analytical themes. The synthesis of the different stakeholder’s data was carefully considered as to whether these were synthesised together or separately depending on the amount of data generated. It was decided there was not sufficient data from different stakeholders to synthesise these separately in order to identify similarities and differences and enable the exploration of different stakeholder experiences and views.

The review aims to provide clear aims, transparent, reproducible methods, rigorous searches, assessment of the validity of the findings, systematic presentation and synthesis of the included studies to provide a reliable source of evidence and minimise bias.^14^ The review protocol was registered with PROSPERO (CRD42021251025). The review was reported in accordance with Enhancing Transparency in Reporting the synthesis of Qualitative research (ENTREQ) guidelines.^15^

## Search strategy

The search strategy was developed using MeSH terms and synonyms for the following three search concepts: (music therapy) AND (children) AND (paediatric palliative care; see search strategy in Supplemental Material). This was developed from studies in similar areas^16–18^ in collaboration with an Academic Liaison Librarian for Health Scien-ces and researchers working in similar clinical areas at the University of York. Electronic health databases were searched systematically in April 2021 and updated in April 2022, these included: PsycINFO, MEDLINE, EMBASE, AMED and CINAHL. Reference searches were also carried out from included studies to identify additional articles, reference lists of relevant literature reviews assessed, and forward citation searching undertaken. The grey literature was searched using an advanced Google Scholar search engine and experts in the field of paediatric palliative care and music therapy were contacted to source any additional literature. Searches followed the ‘7 S’ seven steps approach^19^ which includes: sampling of papers, sources, structured questions, search procedure, search strategies, supplementary strategies and standards for reporting searching.

## Eligibility criteria

Each article was uploaded to the systematic review screening and data extraction tool Covidence^20^ where two reviewers independently screened each article and reviewed these against the inclusion and exclusion criteria detailed below. Disagreements were resolved through discussion and referral to a third reviewer drawn upon where necessary. The SPIDER framework^21^ used in qualitative synthesis was used to develop the inclusion criteria and search strategy (see Table 1).

**Table 1.** Inclusion and exclusion criteria for systematic review using the SPIDER tool.

Concept	Inclusion criteria	Exclusion criteria
Sample	Any of the following: child 0-18 years, parent, family member, music therapist, professionals	Any studies which included the experiences of other participants
Phenomenon of interest	Experience of music therapy in specialist children’s palliative care/children’s hospice setting (either received, delivered or worked alongside)	Any studies which were not focussed on experiences of music therapy in specialist children’s palliative care/children’s hospice settings
Design	Qualitative or mixed methods studies if qualitative data reported separately and could be clearly extracted	Any quantitative studies including studies in which qualitative data had been analysed quantitively
Evaluation	Any qualitative exploration of experiences/perspectives of music therapy delivered by registered Music Therapists	Any quantitative studies described as music in the abstract but not a registered music therapy clinical intervention.
Research type	Peer reviewed journal articles published in English language with no dates imposed. Grey literature	Any studies not published in English language or where full text unavailable

## Data extraction

The data extraction for Qualitative Evidence Synthesis followed the two-stage process identified by Booth et al.^22^ which firstly extracted the contextual details and recorded these in a table of included studies for users of the review to be able to interpret the findings. These included the author/s, publication date, data collection date, country of origin, setting, aims, methodology and methods. Secondly, the findings were extracted from the individual primary studies in the form of quotes from participants, author interpretations, themes, new theory or observational excerpts using the software NiVivo.^9^

## Quality appraisal

Quality appraisal took place for each of the primary qualitative studies identified for inclusion using the Critical Appraisal Skills Programme tool (2020 modified version)^23^ to help understand the strengths and limitations of the methodology of all the included studies and support future recommendations for high quality research. The CQIMG advocate use of the CASP tool, which is widely used for assessment of methodological limitations in health related evidence syntheses.^23^ The CASP appraisal was undertaken independently by both reviewers and studies were attributed a rating of high, medium or low quality to measure methodological quality. Papers were not excluded based on lower methodological quality as it was considered these could still offer credible and applicable data.^24^

## Conducting the synthesis

This review was conducted using Thematic Syntheses,^11^ a well-established approach for synthesising the findings of multiple qualitative studies, facilitating the development of both descriptive and analytical themes from the included papers. Following the steps of thematic synthesis, the text was processed line by line, identifying individual codes which were assessed for their consistency. Coding was undertaken by one reviewer (V.K) and verified for consistency by two additional reviewers (L.F and J.H). After coding each article, the codes were organised into broader groups in order to develop descriptive themes. These descriptive themes were subsequently synthesised further to provide the analytical themes. The Paediatric Palliative Care Research Group Family Advisory Board (a patient and public involvement group) were consulted on all these stages, and these were felt to reflect the experiences of children and families supported by paediatric palliative care.

## Reflexivity

Reflexivity enhances the credibility of findings and fosters a deeper understanding of the subject matter by emphasising the contextual and interconnected relationships between participants and researchers.^25^ In order to ensure quality standards for rigour in qualitative research, researcher views and opinions on experiences of music therapy in paediatric palliative care were carefully considered as possible influences on the review. The review team had diverse professional backgrounds with a range of personal and research experiences and expertise (Music Therapist, Clinical Academic and Applied Health Sciences Researcher). The primary researcher was a music therapist who kept a reflexive diary throughout alongside robust discussions with the other reviewers to ensure rigorous decisions were made in the design and conduct of the review.

## Patient and public involvement

Patient and Public Involvement was central to this research. The Paediatric Palliative Care Research Group Family Advisory Board (a patient and public involvement group) were consulted on the preliminary stages of the review design, analysis of themes and conclusions. The findings resonated with their lived experiences of music therapy with their child/ren in a children’s hospice and they emphasised the importance of capturing the voice of the child in future research. Their input was central in shaping how the themes were described.

## Findings

### Study selection

**Figure 1.**
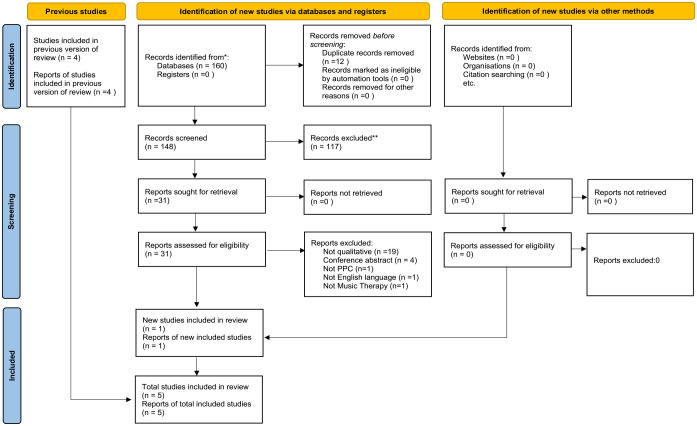
Prisma diagram updated search April 2022.

The full study selection process is presented in Figure 1. Prisma flow diagram updated search.

### Study characteristics

The included studies were published between 2007 and 2021, two were published in Australia, one in Australia and USA, one in the USA and one in Norway. Four were published in peer reviewed journals and one was an unpublished MA thesis (see Table 2). Two of the studies used unspecified interviews, two used semi-structured interviews and one used semi-structured interviews and a focus group as a means of data collection. All studies used phenomenological strategies for analysis.

**Table 2.** Key characteristics of included studies.

Author, Year	Country	Setting	Sample	Participant demographics	Children’s age	Study design	Data collection	Analysis methodology	Aim	Results (themes)
Amadoru, S and McFerran, K 2007	Australia	Children’s Hospice	4 staff members: 2 nurses, 1 carer, 1 hospice manager	Not stated	Not stated	Qualitative	Interviews	Phenomenological strategies	To explore the role of music therapy in children’s hospices from the perception of staff	Communication and expression of feelings and emotions, transcending the ordinary, happiness, better quality of life, desire for more music therapy
Lindenfelser, K.J., Grocke, D. and McFerran, K., 2008	Australia	Community paediatric palliative care	7 bereaved mothers	English speaking, bereavement from 4 months to 4 years. 5 brain tumours, 2 chronic conditions with multiple disabilities	5 months-12 years old	Qualitative	Semi-structured Interviews	Phenomenological strategies	To explore bereaved parents’ experiences of music therapy with their terminally ill child	Altered child and family’s perception of their situation in midst of adversity, significant component of remembrance, multifaceted experience for child and family, enhanced communication and expression
Lindenfelser, K, J Hence, C and McFerran, K 2012	USA and Australia	Children’s hospitals and clinics in USA. Community based paediatric palliative care in Australia	14 families	English speaking Child prognosis greater than one month 9 families USA 5 families Australia. Diagnoses of children include: trisomy 13, various malignancies, metabolic and neurological disorders.	0-14	Mixed methods	Interviews	Phenomenological analysis conducted from in-depth interview data. Data used to evaluate results related to PedsQL Family Impact Module of overall parental quality of life using non-experimental pre-test-post-test design	To explore the role of music therapy in paediatric palliative care and whether this family centred service impacts on overall parental quality of life	Improved child’s physical state, fostered positive experiences, strengths orientated, family centred, facilitated family communication, benefits consistent with improved quality of life
Zuckerman, Z, 2018	USA	Children’s hospital	7 mothers, experienced at least 3 music therapy sessions with their child within last 5 years	English speaking parents of living, terminally ill children All different medical conditions	2–21	Qualitative	Semi-structured interviews	Phenomenological analysis	To explore parents’ experiences when observing or participating in their child’s music therapy sessions in paediatric palliative care	Positive primary and secondary impacts on parents of children receiving palliative care.
Steinhardt, Mortvedt and Trondalen 2021	Norway UK published	Children’s hospital at home	10 families, maximum of 5 sessions over 5 months, 4 nurses	Understanding of Norwegian language, families of paediatric patients with palliative diagnosis.	0-16	Mixed methods	Semi-structured interview/focus group	Phenomenological analysis	To explore the role of music therapy in the hospital-at-home in paediatric palliative care	Isolated yet connected due to music therapy, importance of relationship to music therapist, flexibility and joint music-making, music therapy within hospital-at-home treatment meaningful and valued, family centred care that considers shifting locations, multidisciplinary teamwork valued by staff and families.

Included studies represented the experiences of music therapy from four staff members, seven bereaved mothers, seven mothers (it was not stated whether these were pre or post bereavement) and 24 families (it was not stated which individuals this included). One study interviewed staff members, one interviewed bereaved mothers, two interviewed families and one study interviewed mothers. There was limited information on the demographics of participants, one of the included studies did not state this at all. Where stated, all participants were English speaking staff or parents of life-limited children from Australia or the USA and in one study Norwegian speaking staff or parents from Norway. Bereaved families were between four months and four years bereaved. The health conditions of the children included brain tumours, chronic conditions with multiple disabilities, metabolic and neurological disorders, cancer, severe cardiovascular disease and other undisclosed medical conditions. The ages of the children and young people with life-limiting conditions ranged from five months to 21 years old.

### Quality appraisal results

Each of the studies were subject to quality appraisal using the Critical Appraisal Skills Programme^26^ using a modified version of the tool^23^ for further rigour. One study was low quality, one was low/medium quality, one was medium quality and two were high quality. The category ‘can’t tell’ or ‘somewhat’ was used on numerous occasions as there was insufficient information provided to make a judgement on quality. Table 3 provides details of the CASP appraisal of the included articles.

**Table 3.** CASP Quality appraisal of include studies (modified Long et al.^25^).

CASP question		Study				
		Amadoru, S and McFerran, K 2007	Lindenfelser, K.J., Grocke, D. and McFerran, K., 2008	Lindenfelser, K, J Hence, C and McFerran, K 2012	Zuckerman, Z, 2018	Steinhardt, Mortvedt and Trondalen 2021
Rating		Low	Low/medium	Medium	High	High
1	Was there a clear statement of the aims of the research?	Yes	Yes	Yes	Yes	Yes
2	Is the qualitative methodology appropriate?	Yes	Yes	Yes	Yes	Yes
3	Was the research design appropriate to addresses the aims of the research?	Yes	Yes	Yes	Yes	Yes
4	Are the studies theoretical underpinning clear, consistent and conceptually coherent?	Somewhat	Yes	Yes	Yes	Yes
5	Was the recruitment strategy appropriate to the aims of the research?	No	Yes	Yes	Yes	Yes
6	Was the data collected in a way that addressed the research issue?	Can’t tell	Yes	Yes	Yes	Yes
7	Has the relationship between researcher and participants been adequately considered?	Can’t tell	No	Somewhat	Yes	Can’t tell
8	Have ethical issues been taken into consideration?	No	Yes	Yes	Yes	Yes
9	Was the data analysis sufficiently rigorous?	No	Yes	Somewhat	Yes	Yes
10	Is there a clear statement of findings?	No	Somewhat	Yes	Yes	Yes
11	How valuable is the research? (See below^a^)		A, B	A, B, C	A, B, C	A,B,C

a A. Findings considered in relation to existing research. B. Discussion relating to implications of findings upon practice or policy. C. Identification of areas in which further research is necessary.

### Results of thematic synthesis

Line by line coding was undertaken, 28 codes were developed. Similarities between codes were identified and subsequently grouped into 15 descriptive themes. These descriptive themes were synthesised further to develop five analytic themes which were: emotional and physical reprieve, opportunity for normalised experiences, thriving despite life limited condition, enhance family wellbeing and therapeutic relationship central to outcomes. These are represented in the table below and illustrated in the subsequent narrative using quotes from the included studies (Table 4).

**Table 4.** Relationship between codes, descriptive and analytic themes.

Analytical Themes	Descriptive Themes	Codes
Emotional and physical reprieve	Reprieve Psychological resilience New perspectives	Coping mechanisms Relaxation Energising Distraction from reality Altered perception of life
Thriving despite life limiting condition	Engagement and development Identity reformation Celebration of child	Ability to engage despite LLC Supporting development despite LLC Responses beyond expectations Supporting identity beyond LLC
Opportunity for normalised experiences	Unique medium Ability to meet particular needs of this population Enabling normalised experiences	‘Only’ medium Normality Emotional expression Fun Flexibility Choice and control Meaningful Ongoing benefits Multi-faceted medium
Enhances family wellbeing	Positive impact on family wellbeing Family relationships and experiences supported Continuing bonds supported	Facilitates relationships and communication Increased insight into child Value of witnessing music therapy Value of quality time Quality of life Memory making
Therapeutic relationship central to outcomes	Interpersonal skills of the therapist as important as medium Relationship and reliability integral	Experience of therapist Consistency of therapist Trust in therapist Value of therapeutic relationship

### Emotional and physical reprieve

Emotional and physical reprieve felt in anticipation of, during and after music therapy sessions were reported as central to child and family experiences in all included studies.^7,27–30^ What constituted reprieve was individual for each child and family, ranging from a break in routine,^27,30^ from isolation,^30^ stress and anxiety,^7,28,30^ difficult feelings and conversations,^7,27,28,30^ and medical procedures,^30^ to having an alternative focus provided by somebody else and witnessing their child’s engagement.^7,28,30^ Reprieve was linked to a spectrum of experiences provided from energising to relaxation depending on the individual needs of the child and family in the moment.^29,30^ Emotional and physical reprieve were described as interconnected in many instances; physical relief for the child enabling emotional relief for all the family.^7,27–30^

Many parents reflected on the calming effects of music therapy for their child, suggesting their child may have been experiencing distress or discomfort prior to the sessions and illustrating how music therapy was beneficial in providing relief. . .assisting the family to relax.^29^ p221 (study author)

I have seen the parents revitalise because their child suddenly was really energetic.^30^ p58 (staff)

Music therapy’s capacity to provide ‘relief from the horrible reality’^31^ was significant to family experiences.^7,27,28,30^ Parents described the importance of this medium in providing redirection from negative stimuli,^7,30^ for example when their child was in pain or undergoing a procedure.

Music therapy helps my son away from his pain, to forget the IV’s that he’s getting, the shunts in his head, and being stuck in a bed. I think it takes him to a place other than right there in the hospital room. It makes him think that he’s important and the music takes things away.^7^ p25 (parent)

Trust in the therapist and the medium, its ability to engage the child and enable parents to either leave the room or take a less active role if desired, was described as key to supporting emotional and physical reprieve in families.^7,27,28,30^ Music therapy was characterised as one of the rare times when families felt safe enough to leave the room^7^ and ‘mentally and emotionally escape the hardships of their medical journey’.^7^

I literally never left because he just got a lot of anxiety if I would leave his room. So it was really good for music therapy to come and to have that emotional break for me.^7^ p23 (parent)

Parents depicted music therapy as improving their perception of life and the experiences they were having,^7,28^ providing new perspectives for the whole family.^7,27,28^

Music therapy helped me in that time to think about what I have in my hand – a little angel that was sent to me. And I probably wasn’t thinking about that. I was just trying to find the reason or why this situation was happening. . . . . .So when I listen to what the music therapist is singing, I apply it to my life. So I’m thinking about how beautiful life is and sometimes we just aren’t enjoying what we have in our life.^7^ p27 (parent)

Family members and staff described the reprieve and quality time together which music therapy provided as allowing children and families space to process and reflect on their situation in a safe environment.^7^ This was reported to help families to make sense of their situation,^7^ reflect on what they do have, what their child brings and has taught them^7^ and to become more comfortable,^31^ at peace and resilient.^7^

Parents described music therapy as helping them to understand and find “peace” in the experiences they were having and that music therapy offered a feeling of control that was rare to have in the hospital.^7^ p32 (study author)

It’s great and it helps on so many levels. It helps her makes sense of what is going on but it helps her cope too. It has been a huge blessing.^7^ p26 (parent)

### Opportunity for normalised experiences

Music therapy was depicted as a unique medium,^7,30,28^ with the ability to meet the individual needs of children with life-limiting conditions^7,27–29^ and provide much needed normalised experiences.^7,27,30^ It was highlighted that music therapy was considered rare in its ability to help without causing pain.^7^

For me, [music therapy] is a chance to see, to have somewhat of a normal thing you would do if your child didn’t have the illness and the things that she deals with. You get these moments where it’s like, this is what it could be like or would be like in a way^7^ p29 (parent)

It lifts your heart up a little bit to see your son. Like he’s just getting to be a kid right now and somebody isn’t just seeing him as a patient.^7^ p29 (parent)

This was attributed to the accessibility, adaptability and responsiveness of this model of working to meet the individual needs of these children^7,27–30^ alongside the flexibility of the therapist,^27,30^ providing ‘a bit of normality in a horrible situation’.^28^ Flexibility was a dominant theme which included the framework of sessions, location, length of the session, to the ability to meet children’s individual abilities.^7,30^ Importantly this allowed the music therapy to take place at the time when this was most needed, providing a sense of normality even at the most challenging of times.^7^

. . .following the child’s cues and interests in therapy, while also meeting the child at whatever physical, mental, emotional, and medical level they were at^7^ p31 (study author)

The great thing about music therapy is wherever you’re at, you come to us. There were times in the hospital where my child was able to tinker around on the piano and other times where she was throwing up during music therapy. You’ve seen it all!^7^ p24 (parent)

Music therapy’s ability to provide choice and control was viewed by staff and families as central to normalising children’s experiences^7,27^ as children and families reported to often have little or no control over other aspects of their life.^7,27^

The children have lost so much control over other things in their lives, but they still have this degree of control in music therapy, in when and how they participate and what they choose to share.^27^ p126 (staff)

I couldn’t do a lot for him and the nurses and doctors have to do everything, so I felt kind of helpless. But I can make sure that we stay on top of having music therapy come. I can make sure that continues to happen because I know he benefits from it. So that made me feel better.^7^ p28 (parent)

Importantly music therapy was cited as a space in which children and families experienced fun and meaningful moments together, where ‘something was going to happen’.^30^

### Thriving despite life limited condition

Music therapy was described by families as having a significantly positive impact physically, emotionally and developmentally on their child.^7,27,28,30,31^ The child’s ability to engage, develop, accomplish and thrive in this environment, despite their life limiting condition, dominated all accounts.^7,27,28,30,31^ For parents this was a space where their children were ‘unhindered by the limitations of their child’s illness or disability’.^27^

When their children were otherwise incapacitated or misunderstood, music therapy was a time where parents saw their children thrive and communicate.^7^ p32 (parent)

Music Therapy was something that allowed her to continue being a child.^27^ p124 (staff)

It is worth gold that a music therapist arrives and that she actually manages to vitalize and inspire the child.^30^ p57 (parent)

Importantly it was acknowledged that music therapy was one of the few interventions at the end of the child’s life that allowed children to continue to thrive^28^ and support creativity and expression.

Music therapy was one of the only things at the end of the child’s life that allowed him to continue to thrive as it provided opportunities for expression and creativity.^28^ p338 (parent)

Improved physical states of children were illuminated when accessing music therapy,^7,27,31^ it was felt that this medium was key to ‘relieving distress and increasing overall comfort’^31^ and stimulating responses that occurred premorbidly.^7,27,28,31^

I felt like I was getting a piece of my daughter back every day. And music therapy, I think, sped that along. I got more of her back with music therapy.^7^ p29 (parent)

Links were made to the capacity of music to engage children with limited mobility^7^ and provide opportunities for expression and creativity for these children.^7,28,30,31^

Regardless of the child’s capabilities, he/she was able to respond and engage in music therapy.^28^ p338 (study author)

This in turn supported parental recognition and value of what their child could still do,^7,27,28,31^ creating opportunities to access and witness the healthy aspects of their child,^7,27,28,30,31^ strengthen relationships^7^ and family wellbeing.^7,27,28,30,31^

It gave me great pleasure to see that she could still want to do those sorts of things. Even though her body was giving up her mind was there, "I want to do this, I want to participate in this”.^28^ p341 (parent)

Music therapy was perceived by families and staff as being strengths orientated, a space for the child to thrive, for accomplishments to be recognised and identity to develop beyond that of a child with a life limiting condition.^7,27–30^ Participants highlighted that music therapy provided an opportunity to represent the healthy part of the child ‘crossing the boundaries of disability and allowing them to be more than a sick kid’.^27^ Children’s responses in music therapy were frequently noted as beyond expectations people have of them^27,28^ providing hope and precious moments of celebration in the moment and beyond.^7,27,28,30,31^

He sang for her and he played instruments for her - and this is a baby who has Cerebral Palsy to the extreme of no movement and yet he’s moving his hands and he’s opening his toes. It was like, “Wow, this woman’s performing miracles with our child!”^28^ p342 (parent)

And her first word was singing a song. I mean, no matter how many times we tried to get her to speak, it wasn’t until music therapy that she spoke her first word^7^ p26 (parent)

### Enhance family wellbeing

All articles portrayed music therapy as providing a family centred environment,^7,27–30^ allowing quality time and space^7,30,31^ to help gain a better understanding of one another,^7^ to enjoy witnessing and sharing interactions and experiences^7,27,28,30,31^ and relate and communicate with one another.^27,30^

The sessions provided an activity in which the whole family could participate, allowing them to share musical processes and experience positive emotions.^28^ p341 (study author)

Music therapy offered new opportunities and experiences to families, from providing an avenue for new social connections, to helping parents have the ability to interact with their medically fragile child.^7^ p31 (study author)

These moments of connection were reported to allow ‘the therapeutic benefits of music therapy to extend beyond the child’,^31^ to strengthen family bonds^7,27,28,30,31^ and enhance quality of life.^28,30,31^

When watching music therapy, it feels really good to watch the kids be happy, and just smile, and to participate. That’s what I get out of it, and I assume that the parents would feel the same way, even more so.^32^ p126 (staff)

I am convinced that positive experiences and life quality is really important for the recovery process.^30^ p58 (parent)

Families and staff described the considerable secondary impact derived from witnessing music therapy sessions.^7,28,30–32^

It was for all of us!’ The whole family was gathered. And when he [the child] is happy, we are happy.^30^ p57 (parent)

Simply, if music therapy was helping the child, music therapy was helping the parent.^7^ p32 (study author)

Music therapy was cited as significant not only in the present but into the future, creating precious memories which supported continuing bonds between children and families and family wellbeing.^7,28,31,32^

It makes me happy because I know he is happy. And he loves music. And I can see him trying to move his eyes and he’s telling me, “I love music, mom”. And I just think, later on, I know how I can remember him.^7^ p28 (parent)

Parents discussed how central music therapy had been in developing coping mechanisms and supporting psychological resilience and wellbeing for the whole family in the face of such adversity.^7,28,29^

### Therapeutic relationship central to outcomes

The therapist and therapeutic relationship were highly valued by staff and families and credited to eliciting beneficial outcomes through the ‘personality, skills, adaptability and sensitivity to the children’s needs’^32^ therapists demonstrated.^7,28,30–32^ Trust in the therapist and their expertise and approach^28,30^ allowed families to engage in the work, whether sharing time with their child or enabling much needed reprieve.^7,28,30,32^

The therapeutic relationship was viewed as integral to the outcomes of music therapy, significantly the interpersonal skills of the therapist, their reliability and experience in paediatric palliative care were reported to be as important as the medium.

Music therapists were able to build a relationship of trust with each patient.^7^ p31 (study author)

Feeling comfortable with the therapist^7,28^ was cited as essential due to the close connections shared at such a vulnerable time in family’s lives.^7,28^ Trust was accelerated by the intimate nature of the work,^7^ the therapist’s rapport with the children^27^ and role of ‘confidant’ to parents.^28^ Parents recognised the value of the relationship between the therapist and the child and the bond that continues to develop between stays.^7^

It made it easy for the child and parent to relate, interact, and connect to the music therapist as a confidant who gained trust from the child and family very quickly.^28^ p339 (staff)

Another mother stated that it was great to see her daughter “unfold in music together with another person than with us as parents”, and “I could relax”^30^ p58 (parent)

However, families recognised if the therapist was unable to manage the emotional impact of working with children who are dying^28^ which was attributed to lack of experience and knowledge in paediatric palliative care.^28^ The distinction between children’s and adult’s palliative care was stressed;^28^ experience in paediatric palliative care was described by families as essential to their experience of trust in the therapist and the work^28^ highlighting the importance of the interpersonal skills of the therapist, experience and knowledge in this area and continuity of care.^28^

Parents found it difficult when the music therapist was more familiar working with adults who are dying and appeared to ’struggle’ working with their child who was dying.^28^ p339 (study author)

Parents recognized the value of the therapist-patient relationship and how that bond has continued to foster throughout various inpatient stays at the hospital.^7^ p33 (study author)

Value was particularly placed in the reported rare experience of a professional relationship in their child’s life which provides a positive and beneficial outcome without causing pain or fear.^7^

And it’s not a relationship that involves pain. If you think about it, when you’re in the hospital, so much of what they are doing scares or hurts. So to have somebody coming in that’s not doing those things, that is helping them to get better, is so nice. It is so nice to have somebody that helps that doesn’t have to hurt them while they’re helping him. I love that. I love that about music therapy.^7^ p29 (parent)

The increased emotional vulnerability of parents in sessions was highlighted;^28^ the ability of music to ‘bypass safety gates and just go straight to the emotion’^28^ was experienced as challenging at times.^28^ The potential and responsibility of the therapeutic relationship in music therapy to support parental wellbeing is highlighted here.^28^

## Discussion

### Summary of findings

This is the first synthesis to explore experiences of music therapy in paediatric palliative care. It highlights how music therapy provides children and families with reprieve from the challenges of their lives and opportunities for normalised experiences, where children are seen beyond their life limiting condition and celebrated. Findings shed light on the family centred environment provided by music therapy as facilitating an opportunity for quality family time, to strengthen bonds, develop coping mechanisms and enhance wellbeing pre and post bereavement. The therapeutic relationship, interpersonal skills of the therapist and experience in paediatric palliative care were reported as integral to the outcomes of music therapy. Greater understandings of the experiences of multiple stakeholders emerged, which helps raise awareness of music therapy in paediatric palliative care for policy makers, health professionals and the public in line with recommendations from the World Health Organisation^1^ and to inform future effective service provision, delivery and development.^1^

### What this study adds?

As reflected in this study, previous research has identified that music therapy can help with reduction of anxiety, pain, increasing emotional expression and improving family interactions^7,28^ in paediatric palliative care. This review contributes further knowledge by highlighting stakeholder perspectives of music therapy as a non-medicalised family space, significantly placed within the family centred model of care in paediatric palliative care to support child and family wellbeing,^7,27,28,30,31^ strengthen bonds^7,27,28,31^ and enhance quality of life.^28,31^ This is synonymous with music therapy in adult palliative care literature which reports the ability of music therapy to create a sense of community within the palliative care setting and improve relationships with families and quality of life.^31,33–42^ Findings also mirror those within the wider music therapy literature, which demonstrates how music is used to facilitate relationship building within families or wider groups and foster wellbeing.^18,43–53^ This review goes further by identifying the ability of music therapy to meet the emotional and physical needs of both children and parents and provide repri-eve.^7,27,28,30,31,45,54^ It recognises that parents are often expected to become ‘providers of healthcare for children with very complex needs’,^55^ with higher incidences of physical and mental health challenges reported in mothers^49^ and fathers needs becoming increasingly recognised.^50^ These findings strongly align with the goals of paediatric palliative care from the World Health Organisation, particularly in addressing the relief of pain and suffering, promoting family-centred and holistic care.^1^

Families in paediatric palliative care describe the intense desire for normalcy,^51–53,56^ with illness causing significant disruption to family life. Music therapy was cited as providing access to these much needed normalised experiences^7,27^ in its ability to meet the individual needs of children with life-limiting conditions.^7,27,28,31^ This was attributed to the accessibility and flexibility of the medium^7,28,30^ delivered at the optimum time and space for children and families.^7,28,30^ These themes are reflected in wider music therapy literature,^2,37,57,58^ however this study suggests there is something unique about music therapy for this population in children’s ability not only to still access and respond to this intervention but to thrive in unexpected ways.^7,27,28,30,31,59^ The potential of music therapy to locate the healthy aspects of the child and support their ability to thrive unhindered by their illness or disability^7,27,28,31,59^ is highlighted in this review. This is explored in the wider paediatric palliative care literature,^58^ music therapy in paediatric palliative care literature^2,59,60–63^ and music therapy literature in general paediatrics.^18^ However, this review builds on these ideas and recounts the capacity of music therapy to provide precious and unique moments of engagement and development, supporting children to develop their identity beyond that of their life limiting condition^7,27,28,30,31^ and providing moments of celebration and memory making of particular relevance in this environment.^7,27,28,30,31^ Reframing life experiences by considering alternative views and narratives in the search for meaning is explored in the wider palliative care,^64^ and music therapy in palliative care literature.^35,65^ This study further develops these ideas, illustrating new life perspectives gained in music therapy where families experiences were reframed from losses to strengths, aiding resilience.^7,27,28^ The secondary impact of families witnessing their child’s music therapy was identified as significant. This included the creation of memories and ability to support continuing bonds post bereavement.^7,27–30^

The therapeutic relationship and interpersonal skills of the therapist’s experience in paediatric palliative care were highly valued by stakeholders and viewed as central to eliciting positive outcomes.^7,27,28,31^ The quality of professional relationships in paediatric palliative care are widely recognised as significant for both child and family.^42,53^ Hynson warns that health professionals may underestimate the importance of this relationship, ‘preferring to focus on the biomedical aspects of care’.^53^ Psychoanalytic literature supports the idea that the quality of therapeutic practice is ‘determined by the personal qualities of the therapist and not by the rote enactment of particular rituals or a rigidly structured set of techniques’.^66^ Wider music therapy and paediatric palliative care literature echo the importance of professional relationships in which trust in perceived expertise and the client’s needs are viewed as aiding the creation of a facilitative working environment.^18,60^ The potential and responsibility of the therapeutic relationship in music therapy to support parental emotional wellbeing alongside meeting the child’s needs is highlighted in this review.^28^ A clear distinction between paediatric and adult palliative care was identified; the necessity for experience in paediatric palliative care was described by families as essential to their trust in the therapist and the work.^28^ Families recognised the impact on the work when the therapist was struggling emotionally which was attributed to lack of experience and knowledge in this area.^28^ It could be concluded that goals in music therapy may not be met without effective interpersonal skills of the therapist, a strong therapeutic relationship or appropriate experience and should therefore be prioritised in recruitment and ongoing training and support.

### Strengths/limitations

Key strengths of this review include the systematic approach to identifying relevant studies across five databases, data rich studies with relevance to the research question and the use of thematic synthesis to translate the data into transparent findings. All studies had clear aims and objectives for which qualitative designs were suitable and overall sampling strategies and data collection methods were well designed in the majority of the papers. The use of Patient Public Involvement in this review ensured experiences of parents contributed to shaping the study from design to analysis and highlighted the omission of the voice of the child and the importance of including this in future research. Whilst the review included a breadth of perspectives from staff members, bereaved and pre-bereaved parents and families from Australia, USA and Norway, the number of papers which met the inclusion criteria was only five and represented the experiences of only 18 individuals and 24 families from three different geographical areas. The relationship between the researcher and participants was not adequately considered or possible to tell due to the reporting methods in the majority of the papers and there was not a clear statement of findings in two of the included studies.

## Conclusion

This review explored the experiences of music therapy in paediatric palliative care from multiple stakeholder perspectives. Themes illustrated the unique potential of music therapy to support child and family wellbeing and their ability to thrive. The therapeutic relationship, interpersonal skills of the therapist and experience in paediatric palliative care were viewed as central to these positive outcomes.

Further high-quality research is required to develop an understanding of child and family experiences of music therapy in this setting, positioning these voices as central to service development and provision.

music therapy has made a difference in my life because it’s made a difference in her life more.^7^

## Supplemental Material

sj-docx-1-pmj-10.1177_02692163241230664 – Supplemental material for Experiences of music therapy in paediatric palliative care from multiple stakeholder perspectives: A systematic review and qualitative evidence synthesis Supplemental material, sj-docx-1-pmj-10.1177_02692163241230664 for Experiences of music therapy in paediatric palliative care from multiple stakeholder perspectives: A systematic review and qualitative evidence synthesis by Victoria Kammin, Lorna Fraser, Kate Flemming and Julia Hackett in Palliative Medicine
