# Supplementary material for: Experiences of music therapy in paediatric palliative care from multiple stakeholder perspectives: A systematic review and qualitative evidence synthesis
Source: Palliat Med. 2024 Mar 7;38(3):364–78. doi: 10.1177/02692163241230664 (PMC10955799; doi:10.1177/02692163241230664)
Supplement: sj-docx-1-pmj-10.1177_02692163241230664 – Supplemental material for Experiences of music therapy in paediatric palliative care from multiple stakeholder perspectives: A systematic review and qualitative evidence synthesis [file sj-docx-1-pmj-10.1177_02692163241230664.docx]

**Search strategy**

Taken from:

Barker, M.M., Beresford, B., Bland, M. and Fraser, L.K., 2019. Prevalence and incidence of anxiety and depression among children, adolescents, and young adults with life-limiting conditions: a systematic review and meta-analysis. *JAMA pediatrics*, *173*(9), pp.835-844.

And

Taylor, J., Booth, A., Beresford, B., Phillips, B., Wright, K. and Fraser, L., 2020. Specialist paediatric palliative care for children and young people with cancer: A mixed-methods systematic review. *Palliative medicine*, *34*(6), pp.731-775.

And

Annesley, L., McKeown, E. and Curtis-Tyler, K., 2020. Parents’ perspectives on their children’s music therapy: A synthesis of qualitative literature. *British Journal of Music Therapy*, *34*(1), pp.39-52.

MEDLINE SEARCH TERMS

**Children/young people:**

1. child/ or child, preschool/ or infant/
2. child/ or child, preschool/ or infant.ti,ab.
3. (child$ or children$).ti,ab.
4. Adolescent/
5. Adolescent$.ti,ab.
6. (infant$ or infancy$).ti,ab.
7. (baby or baby$ or babies).ti,ab.
8. (toddler$ or kid or kids).ti,ab.
9. (boy or boys or boyhood or girl or girls or girlhood).ti,ab.
10. (minor or minor$ or schoolchild$).ti,ab.
11. (adolescen$ or juvenil$ or youth$ or teen$ or "under age$" or underage$ or pubescen$).ti,ab.
12. (pediatric$ or paediatric$ or peadiatric$).ti,ab.
13. (young people$ or young person$).ti,ab.

**Palliative care:**

1. exp Palliative Care/
2. exp Terminal Care/
3. palliat$.ti,ab.
4. Palliative Care.ti,ab.
5. terminal care.ti,ab.
6. ("end of life" or "end-of-life").ti,ab.
7. exp Hospices/
8. exp Hospice Care/ ( if doesn’t work - Hospice Care/)
9. Hospice Care.ti,ab.
10. exp Palliative Medicine (if doesn’t work – Palliative Medicine/)
11. Palliative Medicine.ti,ab.
12. "Hospice and Palliative Care Nursing"/ if doesn’t work try exp Hospice and Palliative Care Nursing/
13. "Hospice and Palliative Care Nursing".ti,ab
14. hospice$.ti,ab.

**Music Therapy:**

1. MUSIC THERAPY.mp. or exp Music Therapy/
2. Music therapy*ti.ab
3. music*AND therap*ti.ab
4. Music*adj3therapy*ti.ab
5. (music* and therap*).mp.

**Databases:**

CINAHL

Cochrane Library (Wiley)

MEDLINE

PsycINFO

EMBASE

RILM (Abstracts of Music Literature)

British Journal of Music Therapy

Nordic Journal of Music Therapy

Journal of Music Therapy
